# Calcium platinum aluminium, CaPtAl

**DOI:** 10.1107/S1600536811036749

**Published:** 2011-09-20

**Authors:** Patrice Kenfack Tsobnang, Daniel Fotio, Siméon Ponou, Charles Fon Abi

**Affiliations:** a The University of Yaounde 1, Department of Inorganic Chemistry, Faculty of Sciences, PO Box 812 Yaounde, Cameroon; b The University of Maroua, Department of Chemistry, Ecole Normale Supérieure de Maroua, Cameroon; c The University of Yaounde 1, Department of Chemistry, Ecole Normale Supérieure de Yaoundé, PO Box 47 Yaounde, Cameroon

## Abstract

A preliminary X-ray study of CaPtAl has been reported previously by Hulliger [*J. Alloys Compd* (1993), **196**, 225–228] based on X-ray powder diffraction data without structure refinement. With the present single-crystal X-ray study, we confirm the assignment of the TiNiSi type for CaPtAl, in a fully ordered inverse structure. All three atoms of the asymmetric unit have .*m*. site symmetry. The structure features a _∞_^3^[AlPt] open framework with a fourfold coordination of Pt by Al atoms and *vice versa*. The Ca atoms are located in the large channels of the structure.

## Related literature

For a previous X-ray powder diffraction study of CaPtAl, see: Hulliger (1993 ▶). For related compounds, see: Dascoulidou-Gritner & Schuster (1994 ▶); Merlo *et al.* (1996 ▶). For structural systematics and properties of the TiNiSi structure type, see: Kussmann *et al.* (1998) ▶; Hoffmann & Pöttgen (2001 ▶); Nuspl *et al.* (1996 ▶); Evers *et al.* (1992 ▶). For related compounds of the TiNiSi structure type, see: Ponou & Lidin (2008 ▶); Ponou (2010 ▶); Banenzoué *et al.* (2009 ▶). For atomic radii, see: Pauling (1960 ▶).

## Experimental

### 

#### Crystal data

CaPtAl
*M* *_r_* = 262.15
Orthorhombic, 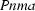

*a* = 7.1581 (14) Å
*b* = 4.2853 (15) Å
*c* = 7.7536 (9) Å
*V* = 237.84 (10) Å^3^
*Z* = 4
Mo *K*α radiation
μ = 61.08 mm^−1^
*T* = 293 K
0.15 × 0.08 × 0.05 mm

#### Data collection

Oxford Diffraction Xcalibur diffractometer with Sapphire3 CCD
Absorption correction: multi-scan (*CrysAlis RED*; Oxford Diffraction, 2007 ▶) *T*_min_ = 0.004, *T*_max_ = 0.047
4413 measured reflections
511 independent reflections
435 reflections with *I* > 2σ(*I*)
*R*_int_ = 0.091

#### Refinement

*R*[*F* ^2^ > 2σ(*F* ^2^)] = 0.028
*wR*(*F* ^2^) = 0.064
*S* = 1.03
511 reflections
20 parameters
Δρ_max_ = 2.61 e Å^−3^
Δρ_min_ = −2.95 e Å^−3^

### 

Data collection: *CrysAlis CCD* (Oxford Diffraction, 2007 ▶); cell refinement: *CrysAlis RED* (Oxford Diffraction, 2007 ▶); data reduction: *CrysAlis CCD*; program(s) used to solve structure: *SHELXS97* (Sheldrick, 2008 ▶); program(s) used to refine structure: *SHELXL97* (Sheldrick, 2008 ▶); molecular graphics: *DIAMOND* (Brandenburg, 1999 ▶); software used to prepare material for publication: *WinGX* (Farrugia, 1999 ▶).

## Supplementary Material

Crystal structure: contains datablock(s) I, global. DOI: 10.1107/S1600536811036749/wm2532sup1.cif

Structure factors: contains datablock(s) I. DOI: 10.1107/S1600536811036749/wm2532Isup2.hkl

Additional supplementary materials:  crystallographic information; 3D view; checkCIF report

## Figures and Tables

**Fig. 1.**
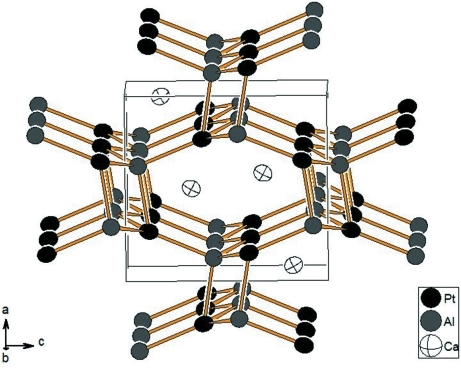
A perspective view of the CaPtAl structure with displacement ellipsoids drawn at the 99% probability level. Ca, Al, and Pt atoms are drawn as white-crossed, grey and black spheres, respectively.

## supplementary crystallographic information

### Comment

A recent re-investigation of the ternary CaAgGe phase from single-crystal X-ray data has revealed a superstructure with a tripling of the *a*-axis of the of the TiNiSi-type basic cell (*i*_3_, minimal isomorphic subgroup of index 3) and a complete ordering of the atomic sites (Ponou & Lidin, 2008; Ponou, 2010). This phase has previously been reported by Merlo *et al.* (1996) in the KHg_2_ structure type with Ag and Ge atoms randomly distributed on the Hg site. Hence, precice single crystals X-ray diffraction measurements is of importance in this family of compounds (Kussmann *et al.*, 1998; Banenzoué *et al.*, 2009) to access both the superstructure and a possible structure inversion with respect to the Ni and Si positions in the three-dimensional framework of fourfold interconnected atoms. Such a structure inversion is observed, for example, in CaGaPt, with Ga atoms at the orginal Ni position and Pt at the original Si position with respect to the prototype TiNiSi structure type (Dascoulidou-Gritner & Schuster, 1994). In 'normal' TiNiSi phases like CaPtGe, Ge occupies the Si position and Pt the Ni position (Evers *et al.*, 1992; Nuspl *et al.*, 1996). Here we report the structure refinement of CaPtAl from single-crystal X-ray diffraction data.

The structure of the title compound was first investigated by Hulliger (1993) from X-ray powder data and assigned to the orthorhombic TiNiSi structure type, however, without a detailed structural refinement. In the present study, the CaPtAl structure was successfully refined as an inverse TiNiSi type. Hence, Ca, Al and Pt atoms are found at Ti, Ni and Si atomic positions, respectively. The origin of the inversion in this structure type is generally ascribed to the relative electronegativity of the framework constituent elements, here Pt and Al. The more electronegative atom (here Pt) is found at the Si position in a strongly distorted tetrahedral (rather pyramidal) coordination whereas the coordination of the less electronegative atom at the Ni position is only slightly distorted from its idealized tetrahedral values (Hoffmann & Pöttgen, 2001). The Pt—Al interactomic distances range from 2.574 (3) Å to 2.675 (3) Å which is comparable with the sum of atomic radii of these elements, *i.e.* 2.62 Å (Al: 1.25 and Pt: 1.37 Å; Pauling, 1960), indicating a covalent character of the bonding. The first coordination sphere of the Ca atoms consists of two counter-tilted Pt_3_Al_3_ hexagons with Ca—Al and Ca—Pt distances ranging from 3.143 (3) Å to 3.489 (3) Å and from 2.978 (2) Å to 3.1179 (16) Å, respectively, also in agreement with the sum of atomic radii (Ca: 1.97 Å).

### Experimental

Single crystals of the title compound, suitable for X-ray diffraction studies, were obtained from a mixture of the elements (Ca ingots (99.5%), Al chunk (99.9%) and Pt pieces (99%), all from ABCR) with a molar ratio Ca:Pt:Al = 2:2:1, by heating in a Nb ampoule at 1253 K for one hour and then slowly cooling at a rate of 6 K/h to room temperature. The product is air-stable, silver-grey with metallic lustre.

### Refinement

The refined unit cell parameters are quite close (slighly lower) to those obtained from a Guinier camera by Hulliger (1993) with *a* = 7.1722 (6), *b* = 4.2885 (4) and *c* = 7.7760 (7) Å. The refinement in the inverse TiNiSi structure model was straightforward. Full/mixed occupancies were checked for all atomic sites by freeing the site occupation factor for each given individual atom, while keeping that of the other atoms fixed. The residual map shows highest peak/deepest hole of 2.61/-2.95 e Å^-2^ at 0.75/0.60 Å from Pt1, respectively.

### Crystal data

**Table:**

CaPtAl	*F*(000) = 444
*M**_r_* = 262.15	*D*_x_ = 7.321 Mg m^−^^3^
Orthorhombic, *P**n**m**a*	Mo *K*α radiation, λ = 0.71073 Å
Hall symbol: -P 2ac 2n	Cell parameters from 2105 reflections
*a* = 7.1581 (14) Å	θ = 2.6–33.7°
*b* = 4.2853 (15) Å	µ = 61.08 mm^−^^1^
*c* = 7.7536 (9) Å	*T* = 293 K
*V* = 237.84 (10) Å^3^	Irregular block, metallic grey
*Z* = 4	0.15 × 0.08 × 0.05 mm

### Data collection

**Table:**

Oxford Diffraction Xcalibur diffractometer with Sapphire3 CCD	511 independent reflections
graphite	435 reflections with *I* > 2σ(*I*)
Detector resolution: 16.1829 pixels mm^-1^	*R*_int_ = 0.091
ω and φ scans	θ_max_ = 33.8°, θ_min_ = 3.9°
Absorption correction: multi-scan (*CrysAlis RED*; Oxford Diffraction, 2007)	*h* = −10→11
*T*_min_ = 0.004, *T*_max_ = 0.047	*k* = −6→6
4413 measured reflections	*l* = −11→12

### Refinement

**Table:**

Refinement on *F*^2^	Primary atom site location: structure-invariant direct methods
Least-squares matrix: full	Secondary atom site location: difference Fourier map
*R*[*F*^2^ > 2σ(*F*^2^)] = 0.028	*w* = 1/[σ^2^(*F*_o_^2^) + (0.0326*P*)^2^] where *P* = (*F*_o_^2^ + 2*F*_c_^2^)/3
*wR*(*F*^2^) = 0.064	(Δ/σ)_max_ < 0.001
*S* = 1.03	Δρ_max_ = 2.61 e Å^−^^3^
511 reflections	Δρ_min_ = −2.95 e Å^−^^3^
20 parameters	Extinction correction: *SHELXL97* (Sheldrick, 2008)
0 restraints	Extinction coefficient: 0.0027 (5)

### Special details

**Table:**

Geometry. All e.s.d.'s (except the e.s.d. in the dihedral angle between two l.s. planes) are estimated using the full covariance matrix. The cell e.s.d.'s are taken into account individually in the estimation of e.s.d.'s in distances, angles and torsion angles; correlations between e.s.d.'s in cell parameters are only used when they are defined by crystal symmetry. An approximate (isotropic) treatment of cell e.s.d.'s is used for estimating e.s.d.'s involving l.s. planes.
Refinement. Refinement of *F*^2^ against ALL reflections. The weighted *R*-factor *wR* and goodness of fit *S* are based on *F*^2^, conventional *R*-factors *R* are based on *F*, with *F* set to zero for negative *F*^2^. The threshold expression of *F*^2^ > σ(*F*^2^) is used only for calculating *R*-factors(gt) *etc*. and is not relevant to the choice of reflections for refinement. *R*-factors based on *F*^2^ are statistically about twice as large as those based on *F*, and *R*- factors based on ALL data will be even larger.

### Fractional atomic coordinates and isotropic or equivalent isotropic displacement parameters (Å^2^)

**Table:**

	*x*	*y*	*z*	*U*_iso_*/*U*_eq_	
Pt1	0.78928 (5)	0.25	0.38355 (4)	0.01029 (15)	
Al1	0.1446 (5)	0.25	0.4346 (4)	0.0103 (6)	
Ca1	0.4796 (3)	0.75	0.3234 (2)	0.0106 (4)	

### Atomic displacement parameters (Å^2^)

**Table:**

	*U*^11^	*U*^22^	*U*^33^	*U*^12^	*U*^13^	*U*^23^
Pt1	0.00775 (19)	0.0118 (2)	0.0113 (2)	0	0.00104 (12)	0
Al1	0.0087 (13)	0.0127 (15)	0.0094 (11)	0	0.0001 (11)	0
Ca1	0.0088 (8)	0.0129 (10)	0.0102 (8)	0	−0.0011 (6)	0

### Geometric parameters (Å, °)

**Table:**

Pt1—Al1^i^	2.574 (3)	Al1—Ca1^xi^	3.160 (3)
Pt1—Al1^ii^	2.6084 (17)	Al1—Ca1^ix^	3.160 (3)
Pt1—Al1^iii^	2.6084 (17)	Al1—Ca1^ii^	3.280 (4)
Pt1—Al1^iv^	2.675 (3)	Al1—Ca1	3.329 (3)
Pt1—Ca1^ii^	2.978 (2)	Al1—Ca1^vi^	3.329 (3)
Pt1—Ca1^v^	3.0037 (14)	Ca1—Pt1^ii^	2.978 (2)
Pt1—Ca1^iv^	3.0037 (14)	Ca1—Pt1^ix^	3.0037 (14)
Pt1—Ca1^vi^	3.1179 (16)	Ca1—Pt1^xii^	3.0037 (14)
Pt1—Ca1	3.1179 (16)	Ca1—Pt1^xiii^	3.1179 (16)
Pt1—Ca1^vii^	3.791 (2)	Ca1—Al1^xiv^	3.143 (3)
Al1—Pt1^viii^	2.574 (3)	Ca1—Al1^iv^	3.160 (3)
Al1—Pt1^ii^	2.6084 (17)	Ca1—Al1^xv^	3.160 (3)
Al1—Pt1^iii^	2.6084 (17)	Ca1—Al1^ii^	3.280 (4)
Al1—Pt1^ix^	2.675 (3)	Ca1—Al1^xiii^	3.329 (3)
Al1—Ca1^x^	3.143 (3)	Ca1—Ca1^xvi^	3.489 (3)
			
Al1^i^—Pt1—Al1^ii^	74.79 (9)	Ca1^xi^—Al1—Ca1	122.80 (9)
Al1^i^—Pt1—Al1^iii^	74.79 (8)	Ca1^ix^—Al1—Ca1	70.67 (4)
Al1^ii^—Pt1—Al1^iii^	110.46 (10)	Ca1^ii^—Al1—Ca1	63.73 (7)
Al1^i^—Pt1—Al1^iv^	121.62 (9)	Pt1^viii^—Al1—Ca1^vi^	132.22 (7)
Al1^ii^—Pt1—Al1^iv^	124.66 (5)	Pt1^ii^—Al1—Ca1^vi^	122.54 (12)
Al1^iii^—Pt1—Al1^iv^	124.66 (5)	Pt1^iii^—Al1—Ca1^vi^	58.71 (5)
Al1^i^—Pt1—Ca1^ii^	121.43 (7)	Pt1^ix^—Al1—Ca1^vi^	58.83 (6)
Al1^ii^—Pt1—Ca1^ii^	72.83 (7)	Ca1^x^—Al1—Ca1^vi^	116.88 (8)
Al1^iii^—Pt1—Ca1^ii^	72.83 (7)	Ca1^xi^—Al1—Ca1^vi^	70.67 (4)
Al1^iv^—Pt1—Ca1^ii^	116.96 (8)	Ca1^ix^—Al1—Ca1^vi^	122.80 (9)
Al1^i^—Pt1—Ca1^v^	68.52 (5)	Ca1^ii^—Al1—Ca1^vi^	63.73 (7)
Al1^ii^—Pt1—Ca1^v^	142.42 (8)	Ca1—Al1—Ca1^vi^	80.12 (9)
Al1^iii^—Pt1—Ca1^v^	67.70 (7)	Pt1^ii^—Ca1—Pt1^ix^	96.57 (5)
Al1^iv^—Pt1—Ca1^v^	71.52 (6)	Pt1^ii^—Ca1—Pt1^xii^	96.57 (5)
Ca1^ii^—Pt1—Ca1^v^	134.49 (3)	Pt1^ix^—Ca1—Pt1^xii^	91.01 (6)
Al1^i^—Pt1—Ca1^iv^	68.52 (5)	Pt1^ii^—Ca1—Pt1^xiii^	110.21 (5)
Al1^ii^—Pt1—Ca1^iv^	67.70 (7)	Pt1^ix^—Ca1—Pt1^xiii^	153.20 (7)
Al1^iii^—Pt1—Ca1^iv^	142.42 (8)	Pt1^xii^—Ca1—Pt1^xiii^	84.96 (3)
Al1^iv^—Pt1—Ca1^iv^	71.52 (6)	Pt1^ii^—Ca1—Pt1	110.21 (5)
Ca1^ii^—Pt1—Ca1^iv^	134.49 (3)	Pt1^ix^—Ca1—Pt1	84.96 (3)
Ca1^v^—Pt1—Ca1^iv^	91.01 (6)	Pt1^xii^—Ca1—Pt1	153.20 (7)
Al1^i^—Pt1—Ca1^vi^	136.51 (3)	Pt1^xiii^—Ca1—Pt1	86.82 (5)
Al1^ii^—Pt1—Ca1^vi^	140.75 (8)	Pt1^ii^—Ca1—Al1^xiv^	123.30 (9)
Al1^iii^—Pt1—Ca1^vi^	69.23 (7)	Pt1^ix^—Ca1—Al1^xiv^	50.15 (4)
Al1^iv^—Pt1—Ca1^vi^	65.60 (6)	Pt1^xii^—Ca1—Al1^xiv^	50.15 (4)
Ca1^ii^—Pt1—Ca1^vi^	69.79 (5)	Pt1^xiii^—Ca1—Al1^xiv^	110.15 (6)
Ca1^v^—Pt1—Ca1^vi^	75.66 (3)	Pt1—Ca1—Al1^xiv^	110.15 (6)
Ca1^iv^—Pt1—Ca1^vi^	137.12 (3)	Pt1^ii^—Ca1—Al1^iv^	136.43 (5)
Al1^i^—Pt1—Ca1	136.51 (3)	Pt1^ix^—Ca1—Al1^iv^	49.29 (6)
Al1^ii^—Pt1—Ca1	69.23 (7)	Pt1^xii^—Ca1—Al1^iv^	108.36 (7)
Al1^iii^—Pt1—Ca1	140.74 (8)	Pt1^xiii^—Ca1—Al1^iv^	107.15 (8)
Al1^iv^—Pt1—Ca1	65.60 (6)	Pt1—Ca1—Al1^iv^	50.44 (6)
Ca1^ii^—Pt1—Ca1	69.79 (5)	Al1^xiv^—Ca1—Al1^iv^	59.91 (8)
Ca1^v^—Pt1—Ca1	137.12 (3)	Pt1^ii^—Ca1—Al1^xv^	136.43 (5)
Ca1^iv^—Pt1—Ca1	75.66 (3)	Pt1^ix^—Ca1—Al1^xv^	108.36 (7)
Ca1^vi^—Pt1—Ca1	86.82 (5)	Pt1^xii^—Ca1—Al1^xv^	49.29 (6)
Al1^i^—Pt1—Ca1^vii^	55.28 (7)	Pt1^xiii^—Ca1—Al1^xv^	50.44 (6)
Al1^ii^—Pt1—Ca1^vii^	55.55 (5)	Pt1—Ca1—Al1^xv^	107.15 (8)
Al1^iii^—Pt1—Ca1^vii^	55.55 (5)	Al1^xiv^—Ca1—Al1^xv^	59.91 (8)
Al1^iv^—Pt1—Ca1^vii^	176.90 (8)	Al1^iv^—Ca1—Al1^xv^	85.38 (9)
Ca1^ii^—Pt1—Ca1^vii^	66.144 (17)	Pt1^ii^—Ca1—Al1^ii^	95.37 (7)
Ca1^v^—Pt1—Ca1^vii^	106.42 (4)	Pt1^ix^—Ca1—Al1^ii^	132.66 (3)
Ca1^iv^—Pt1—Ca1^vii^	106.42 (4)	Pt1^xii^—Ca1—Al1^ii^	132.66 (3)
Ca1^vi^—Pt1—Ca1^vii^	116.41 (5)	Pt1^xiii^—Ca1—Al1^ii^	48.04 (3)
Ca1—Pt1—Ca1^vii^	116.41 (5)	Pt1—Ca1—Al1^ii^	48.04 (3)
Pt1^viii^—Al1—Pt1^ii^	105.21 (8)	Al1^xiv^—Ca1—Al1^ii^	141.34 (12)
Pt1^viii^—Al1—Pt1^iii^	105.21 (8)	Al1^iv^—Ca1—Al1^ii^	93.19 (5)
Pt1^ii^—Al1—Pt1^iii^	110.46 (10)	Al1^xv^—Ca1—Al1^ii^	93.19 (5)
Pt1^viii^—Al1—Pt1^ix^	103.93 (10)	Pt1^ii^—Ca1—Al1	48.46 (5)
Pt1^ii^—Al1—Pt1^ix^	115.36 (8)	Pt1^ix^—Ca1—Al1	49.65 (5)
Pt1^iii^—Al1—Pt1^ix^	115.36 (8)	Pt1^xii^—Ca1—Al1	105.70 (8)
Pt1^viii^—Al1—Ca1^x^	82.41 (9)	Pt1^xiii^—Ca1—Al1	156.29 (8)
Pt1^ii^—Al1—Ca1^x^	62.14 (6)	Pt1—Ca1—Al1	91.78 (5)
Pt1^iii^—Al1—Ca1^x^	62.14 (6)	Al1^xiv^—Ca1—Al1	92.55 (6)
Pt1^ix^—Al1—Ca1^x^	173.66 (14)	Al1^iv^—Ca1—Al1	89.81 (6)
Pt1^viii^—Al1—Ca1^xi^	62.19 (7)	Al1^xv^—Ca1—Al1	150.39 (11)
Pt1^ii^—Al1—Ca1^xi^	165.23 (11)	Al1^ii^—Ca1—Al1	116.27 (7)
Pt1^iii^—Al1—Ca1^xi^	81.55 (4)	Pt1^ii^—Ca1—Al1^xiii^	48.46 (5)
Pt1^ix^—Al1—Ca1^xi^	63.96 (6)	Pt1^ix^—Ca1—Al1^xiii^	105.70 (8)
Ca1^x^—Al1—Ca1^xi^	120.09 (8)	Pt1^xii^—Ca1—Al1^xiii^	49.65 (5)
Pt1^viii^—Al1—Ca1^ix^	62.19 (7)	Pt1^xiii^—Ca1—Al1^xiii^	91.78 (5)
Pt1^ii^—Al1—Ca1^ix^	81.55 (4)	Pt1—Ca1—Al1^xiii^	156.29 (8)
Pt1^iii^—Al1—Ca1^ix^	165.23 (11)	Al1^xiv^—Ca1—Al1^xiii^	92.55 (6)
Pt1^ix^—Al1—Ca1^ix^	63.96 (6)	Al1^iv^—Ca1—Al1^xiii^	150.39 (10)
Ca1^x^—Al1—Ca1^ix^	120.09 (8)	Al1^xv^—Ca1—Al1^xiii^	89.81 (6)
Ca1^xi^—Al1—Ca1^ix^	85.38 (9)	Al1^ii^—Ca1—Al1^xiii^	116.27 (7)
Pt1^viii^—Al1—Ca1^ii^	153.94 (11)	Al1—Ca1—Al1^xiii^	80.12 (9)
Pt1^ii^—Al1—Ca1^ii^	62.73 (6)	Pt1^ii^—Ca1—Ca1^xvi^	56.99 (5)
Pt1^iii^—Al1—Ca1^ii^	62.73 (6)	Pt1^ix^—Ca1—Ca1^xvi^	153.54 (10)
Pt1^ix^—Al1—Ca1^ii^	102.13 (11)	Pt1^xii^—Ca1—Ca1^xvi^	91.10 (3)
Ca1^x^—Al1—Ca1^ii^	71.53 (6)	Pt1^xiii^—Ca1—Ca1^xvi^	53.22 (4)
Ca1^xi^—Al1—Ca1^ii^	131.96 (7)	Pt1—Ca1—Ca1^xvi^	104.19 (8)
Ca1^ix^—Al1—Ca1^ii^	131.96 (7)	Al1^xiv^—Ca1—Ca1^xvi^	140.93 (5)
Pt1^viii^—Al1—Ca1	132.22 (7)	Al1^iv^—Ca1—Ca1^xvi^	151.87 (11)
Pt1^ii^—Al1—Ca1	58.71 (5)	Al1^xv^—Ca1—Ca1^xvi^	92.82 (5)
Pt1^iii^—Al1—Ca1	122.54 (12)	Al1^ii^—Ca1—Ca1^xvi^	58.83 (7)
Pt1^ix^—Al1—Ca1	58.83 (6)	Al1—Ca1—Ca1^xvi^	104.61 (9)
Ca1^x^—Al1—Ca1	116.88 (8)	Al1^xiii^—Ca1—Ca1^xvi^	57.44 (6)

Symmetry codes: (i) *x*+1, *y*, *z*; (ii) −*x*+1, −*y*+1, −*z*+1; (iii) −*x*+1, −*y*, −*z*+1; (iv) *x*+1/2, *y*, −*z*+1/2; (v) *x*+1/2, *y*−1, −*z*+1/2; (vi) *x*, *y*−1, *z*; (vii) −*x*+3/2, −*y*+1, *z*+1/2; (viii) *x*−1, *y*, *z*; (ix) *x*−1/2, *y*, −*z*+1/2; (x) −*x*+1/2, −*y*+1, *z*+1/2; (xi) *x*−1/2, *y*−1, −*z*+1/2; (xii) *x*−1/2, *y*+1, −*z*+1/2; (xiii) *x*, *y*+1, *z*; (xiv) −*x*+1/2, −*y*+1, *z*−1/2; (xv) *x*+1/2, *y*+1, −*z*+1/2; (xvi) −*x*+1, −*y*+2, −*z*+1.

## References

[bb1] Banenzoué, C., Ponou, S. & Lambi, J. N. (2009). *Acta Cryst.* E**65**, i90. 10.1107/S1600536809048454 PMC2971942 21578545

[bb2] Brandenburg, K. (1999). *DIAMOND* Crystal Impact GbR, Bonn, Germany.

[bb3] Dascoulidou-Gritner, K. & Schuster, H. U. (1994). *Z. Anorg. Allg. Chem.* **620**, 1151–1156.

[bb4] Evers, J., Oehlinger, G., Polborn, K. & Sendlinger, B. (1992). *J. Alloys Compd*, **182**, L23–L29.

[bb5] Farrugia, L. J. (1999). *J. Appl. Cryst.* **32**, 837–838.

[bb6] Hoffmann, R.-D. & Pöttgen, R. (2001). *Z. Kristallogr.* **216**, 127–145.

[bb7] Hulliger, F. (1993). *J. Alloys Compd*, **196**, 225–228.

[bb8] Kussmann, D., Hoffmann, R.-D. & Pöttgen, R. (1998). *Z. Anorg. Allg. Chem.* **624**, 1727–1735.

[bb9] Merlo, F., Pani, M. & Fornasini, M. L. (1996). *J. Alloys Compd*, **232**, 289–295.

[bb10] Nuspl, G., Polborn, K., Evers, J., Landrum, G. A. & Hoffmann, R. (1996). *Inorg. Chem.* **35**, 6922–6932. 10.1021/ic9602557 11666868

[bb11] Oxford Diffraction (2007). *CrysAlis CCD* and *CrysAlis RED* Oxford Diffraction Ltd, Abingdon, England

[bb12] Pauling, L. (1960). *The Nature of the Chemical Bond*, 3rd ed. Ithaca: Cornell University Press.

[bb13] Ponou, S. (2010). *Eur. J. Inorg. Chem.*, pp. 4139–4147.

[bb14] Ponou, S. & Lidin, S. (2008). *Z. Kristallogr. New Cryst. Struct.* **223**, 329–330.

[bb15] Sheldrick, G. M. (2008). *Acta Cryst.* A**64**, 112–122. 10.1107/S0108767307043930 18156677

